# Associations of Mood on Objective and Subjective Cognitive Complaints in Persons Living with HIV/AIDS

**DOI:** 10.16966/2380-5536.146

**Published:** 2018-01-09

**Authors:** Moka Yoo-Jeong, Ashley Anderson, AKM Fazlur Rahman, Maya Baumann, Jade McBroom, Drenna Waldrop-Valverde

**Affiliations:** 1 Nell Hodgson Woodruff School of Nursing, Emory University, Atlanta, USA; 2 Department of Biostatistics, The University of Alabama at Birmingham, Birmingham, USA

**Keywords:** Mood, Depression, Cognitive function, Cognitive complaints, HIV

## Abstract

Healthcare workers commonly rely on patient self-report to identify problems with cognitive functioning among Persons Living with HIV (PLWH). Self-reported cognitive complaints may not accurately reflect objective cognitive performance and may be obscured by co-occurring depression. The purpose of the current study was to examine the relationships among depression, subjective cognitive complaints, and objective cognitive performance in PLWH using measures easily administered by healthcare workers. Particularly, this study assessed the association between subjective cognitive complaints (MOS-HIV) and objective cognitive performance (mHDS) using a simple screening tool, as well as whether depressive symptoms (CES-D 10) moderated this relationship. This was a secondary data analysis of a parent study that enrolled participants (N=207) from outpatient HIV clinics in Florida between 2009 and 2011. Most participants identified themselves as African American (82.6%) and heterosexual (81.6%). Almost half of the participants were male (46.4%). Fifty-one percent of participants had a score of 10 or greater on CES-D, indicating clinical depression. This study found no association between subjective and objective cognitive measures; depressive symptoms exhibited no moderating effect on the relationship between subjective cognitive complaints and objective cognitive performance. Depressive symptoms were significantly associated with subjective perceptions of cognitive ability. Results suggest that subjective cognitive complaints may be an inadequate tool for identifying objective cognitive impairments among PLWH. Additionally, treatment of depressive symptoms may help alleviate subjective cognitive complaints.

## Introduction

Despite the use of current combination antiretroviral therapy, cognitive impairment is one of the most common clinical problems in persons living with HIV PLWH [1]. Although the rates of more serious forms of HIV-associated neurocognitive disorders, such as HIV-associated dementia, have been substantially reduced since the advent of antiretroviral therapy, PLWH continue to experience milder forms of HIV-associated neurocognitive disorders, including minor neurocognitive disorder and asymptomatic neurocognitive impairment. It is estimated that the prevalence of minor neurocognitive disorder and asymptomatic neurocognitive impairment among PLWH ranges from 12% to 33% respectively [1]. The cognitive domains commonly affected in PLWH include information processing, attention/working memory, learning and memory, and executive function [2,3]. These cognitive deficits may negatively affect PLWH’s quality of life, medication adherence, and retention in HIV care [4,5].

Healthcare workers often depend on patient self-report to detect and address cognitive impairment. Yet, patient self-report may not accurately reflect an individual’s objective neuropsychological performance. While some studies have found a relationship between subjective complaints of cognitive deficits and objective neuropsychological performance [6–10], other studies have failed to find a significant relationship [11–13]. These inconsistencies between subjective complaints of cognition and objective neuropsychological performance raise concerns about reliance on self-reported cognitive complaints to guide treatment decisions.

Further complicating cognitive problems is the issue of co-occurring symptoms of depression. Depression, one of the most prevalent mental disorders in PLWH [14], involves affective, somatic, and cognitive symptoms [15]. A recent meta-analysis of 14 studies shows that increased severity of depression is associated with decreased neuropsychological performance [16]. Studies suggest that depressed mood may also influence subjective cognitive complaints among PLWH [7,10,17–20]. However, many of the studies that link cognitive function to depressive symptoms were conducted during the pre-antiretroviral therapy era when the nature and prevalence of cognitive deficits differed from those seen in PLWH on contemporary antiretroviral therapy regimens.

Additionally, these studies included extensive neurocognitive testing that while preferred for hypothesis testing, are not feasible as a quick and easily administered test by healthcare staff. Given that frontline healthcare workers are typically the only available providers to assess and advocate for cognitive issues, simple and easy-to-administer approaches that help to differentiate mood and cognitive symptoms are essential. Moreover, early treatment of HIV with antiretroviral therapy may reduce the development and progression of cognitive deficits [1]. Therefore, accurate and early identification of symptoms may have important implications for preventing the development of HIV-associated neurocognitive disorders.

The purpose of the current study was to examine the relationships among depression, subjective cognitive complaints, and objective neuropsychological performance in PLWH using accessible, simple, and short measures that could be administered easily by healthcare workers. We hypothesize that depression would be associated with subjective cognitive complaints but not with objective neuropsychological performance. We first assessed whether subjective cognitive complaints and objective neuropsychological performance were associated with one another. Second, we tested the association of depressive symptoms and subjective cognitive functioning on an objective measure of cognition and explored if depressive symptoms moderate the association between subjective and objective cognitive functioning.

## Methods

This is a secondary data analysis from a prospective study that examined the effects of health literacy, cognitive impairment, provider relationships, and social support on medical visit adherence. The parent study was approved by the University of Miami Institutional Review Board. HIV-positive participants were recruited through flyers, word-of-mouth, and referrals from clinic personnel between August 2009 and May 2011. All participants were patients from the Special Immunology Clinics affiliated with the University of Miami, Jackson Memorial Hospital, and the Borinquen Community Health Center in South Florida. These public hospitals and clinics serve mostly African American and Hispanic persons from minority communities in and surrounding the city of Miami. Inclusion criteria from the parent study were as follows: attended at least one routine medical care appointment 28 weeks prior to baseline, not concurrently enrolled in a pharmacological trial for antiretroviral therapy, English-speaking, no psychotic illness (e.g., schizophrenia, bipolar disorder), and no history of loss-of-consciousness greater than 30 minutes. Participant eligibility was confirmed *via* medical records.

This secondary analysis used data from the parent study’s baseline assessment. Baseline study visits, occurring within 2 weeks of initial recruitment and screening, were held face-to-face in a private office on the University of Miami campus located in a building separate from the recruiting clinics. Baseline assessments were conducted by trained research staff supervised by the study neuropsychologist after obtaining the informed consent.

### Measures

#### Demographics

All participants completed a general questionnaire assessing basic socio-demographic information (age, gender, race, education, income, employment, housing, time since HIV diagnosis).

#### HIV biomarkers

Current CD4 T cell count, plasma HIV-RNA, and % undetectable (<50 copies/mL), AIDS status, current antiretroviral therapy use, and duration of treatment were collected from the electronic medical records.

#### Depression

The Center for Epidemiological Studies-Depression 10 (CES-D 10) was used to assess depressive symptoms. CES-D 10 is a short, 10-item Likert scale questionnaire that was derived from the CES-D 20 [21,22]. Item responses range from 0 (“rarely or none of the time”) to 3 (“all of the time”). Scores range from 0 to 30. A score of 10 or more indicates significant depressive symptoms. The CES-D 10 has high internal consistency and good validity across a variety of populations [23,24]. Within this study, the internal reliability of the CES-D 10 was adequate (α=0.709).

#### Subjective Cognitive Complaints

The Medical Outcomes Study HIV Health Survey (MOS-HIV) was administered to evaluate various self-reported health indicators among PLWH [25]. This instrument contains ten subscales that measure general health, physical function, role function, social function, cognitive function, pain, mental health, fatigue, health distress, and quality of life. This secondary analysis focused on the 4-item cognitive subscale, which assesses personal perceptions of reasoning, recall, attention, and concentration. Item responses range from 1 (“All of the time”) to 6 (“None of the time”), with higher scores indicating better cognitive functioning. Previous research indicates that the overall instrument possesses adequate content/construct validity, high internal consistency, and adequate test-retest reliability [25–27]. Within the parent study, internal reliability of the overall MOS-HIV instrument (α=0.91) and the cognitive assessment subscale (α=0.86) were good.

#### Objective Assessment of Cognitive Ability

The 3-item modified HIV Dementia Scale (mHDS) was administered as a measure of objective neuropsychological performance [28]. The full HDS includes an item that requires specialized training (i.e., the anti-saccadic eye movement test). However, the modified version does not contain this item, thus eliminating the need for a highly-trained neurologist. The mHDS was designed to identify subcortical dementias by evaluating and timing psychomotor speed (writing the alphabet), memory (word recall), and visuo-construction by drawing a cube [28]. The maximum attainable score is 12 and individuals with scores less than 10 should be evaluated for possible dementia. Scores less than 7.5 indicate likely HIV-associated dementia. This instrument has been shown to be sensitive in identifying severe cognitive deficits associated with HIV-associated dementia [28]. Since this is an objective, performance-based measure, internal reliability measures are not appropriate.

### Statistical analysis

Descriptive statistics were performed for sociodemographic and clinical characteristics. Correlation analysis was performed to assess the association between subjective cognitive complaints (MOS-HIV) and objective neuropsychological performance (mHDS). Multiple linear regression was used to examine the effects of depressive symptoms and subjective assessment of cognition on objective neuropsychological performance. PROCESS module of SPSS version 24 was employed to test the moderating effect of depressive symptoms on the association between subjective cognitive complaints and objective neuropsychological performance. Prior to regression analysis, an initial stepwise variable selection was performed. The final multiple linear regression analysis was adjusted for the covariates (age, gender, and education) with *p*-value less than or equal to 0.05. Residuals plots and histograms were constructed to assess the normality assumption of the regression model. Since both subjective and objective measures of cognition (MOS-HIV and mHDS) were left-skewed, Box-Cox transformations were performed prior to all analyses. All statistical procedures were performed using statistical package *R* 3.2.2 and IBM SPSS for Windows version 24.

## Results

A total of 210 participants were enrolled in the parent study. For our statistical analysis, we used data from 207 subjects with complete observations on the variables of interest.

Demographic and clinical characteristics are summarized in table 1. Most participants identified themselves as African American (82.6%) and heterosexual (81.6%). Over half of the participants reported that they were never-married/single, 29% reported that they were divorced/separated, and over 9% reported that they were married. Ninety-one percent of the participants reported that they were unemployed and nearly one out of five (18.8%) reported not having a place to live in the past 7 months. The age of participants ranged from 24 to 70 years with a mean (Standard Deviation [SD]) age of 47.02 (7.43) years. Forty percent of participants were aged 50 or older and the average number of years of completed education was 11.07 (SD=2.10). The average time since initial HIV diagnosis was almost 13 years. The mean (SD) CES-D 10 score was 10.72 (6.63). The mean (SD) score for the subjective cognitive complaints, MOS-HIV cognitive subscale, was 73.10 (19.31). The mean (SD) score for the objective neuropsychological performance, mHDS, was 7.30 (3.14) with 25% of participants scoring 4.5 or lower. These scores suggest that at least half of the sample may be at risk for cognitive impairment and would benefit from a comprehensive assessment.

We explored whether the scores of subjective cognitive complaints (MOS-HIV), objective neuropsychological performance (mHDS), and depression (CES-D10) differed by unemployment status and homelessness. Results show that there were no significant differences of the mean scores of MOS-HIV (77.0 *vs*. 72.5, *p*=0.34) and CES-D10 (9.5 *vs*. 10.9, *p*=0.38) by unemployment status. However, the mean score of mHDS differed by unemployment status (7.67 *vs*. 6.33, *p*=0.034). The mean scores of CES-D10 (13.4 *vs*. 10.2, *p*=0.006) and of mHDS (5.7 *vs*. 6.63, *p*=0.05) were significantly different by homelessness status. The mean score of MOS-HIV (68.7 *vs*. 73.9, *p*=0.13) was not significantly different by homelessness status.

**Figure 1:**
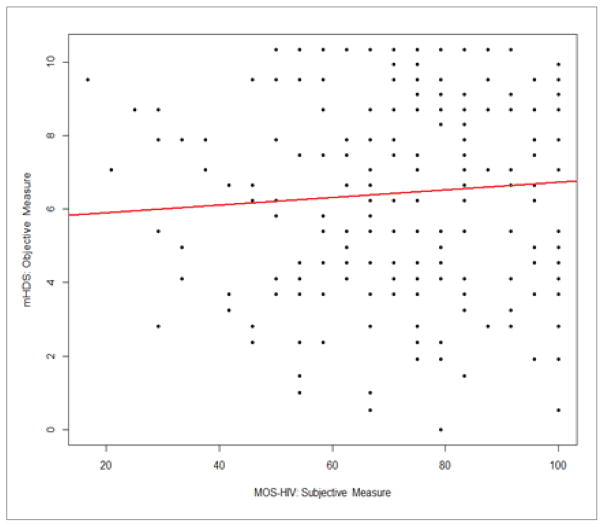
Correlation between subjective cognitive complaints (MOS-HIV) and objective neuropsychological performance (mHDS) **Note:** MOS-HIV=Medical Outcomes Survey-HIV Cognitive Subscale; mHDS=Modified HIV-Dementia Scale

**Figure 2:**
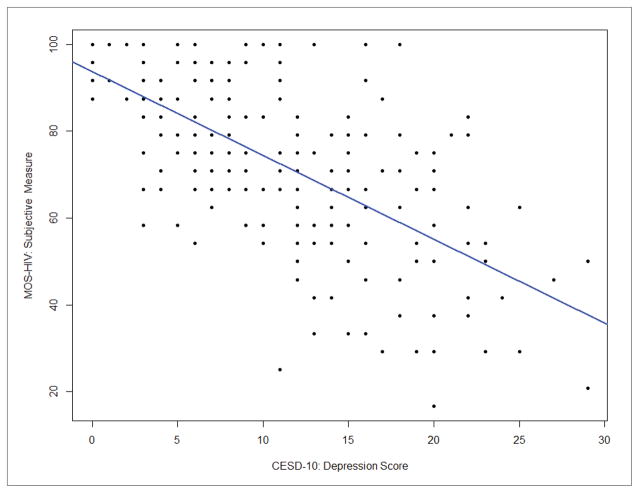
Correlation between subjective cognitive complaints and depression (CES-D10) **Note:** CES-D 10=Center for Epidemiological Studies-Depression 10; MOS-HIV=Medical Outcomes Survey-HIV Cognitive Subscale

**Figure 3:**
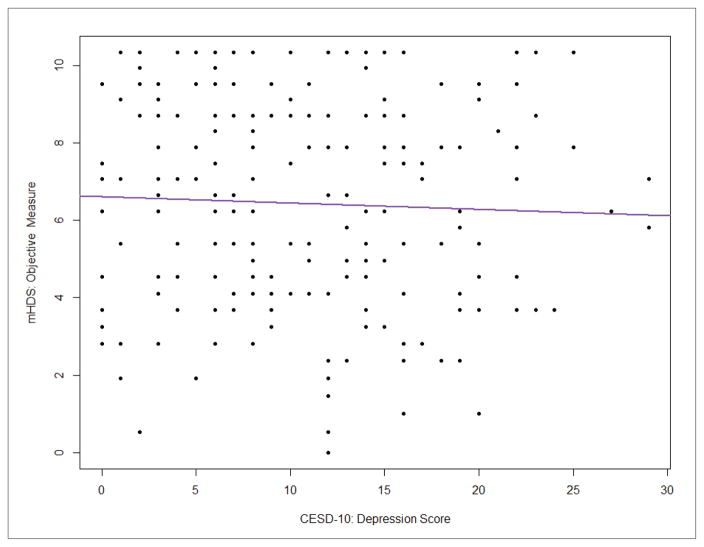
Correlation between objective neuropsychological performance (mHDS) and depression (CES-D10) **Note:** CES-D 10=Center for Epidemiological Studies-Depression 10; mHDS=Modified HIV-Dementia Scale

Correlation analysis was used to test bivariate associations (Table 2). This analysis shows that objective neuropsychological performance was not associated with subjective cognitive complaints (*r*=0.076; Confidence Interval [CI]: −0.06, 0.21; *p*=0.28) or depression (*r*=−0.04; CI: −0.18, 0.10; *p*=0.56). Results also demonstrate that subjective cognitive complaints had a moderate inverse association with depression in our sample (*r*=−0.66, CI: −0.73, −0.57; *p*<0.0001). Because both measures of depression (CES-D 10) and subjective cognitive complaints (MOS-HIV cognitive subscale) include a similar item (i.e., concentration problems), we completed two separate analyses with and without the similar item on the CES-D 10. The result of the correlation analysis between MOS-HIV and CES-D 10 was not significantly different from the analysis without the similar item on the CES-D 10 scale (*r*=−0.63, CI: −0.71, −0.54), and the multiple linear regression using either full CES-D 10 or revised CES-D 10 showed no major difference (both models had R^2^=0.12 with only slight changes to regression coefficients), controlled for other covariates; herein we only include results using the full CES-D 10 scale. The results of the main correlations are plotted in figures 1–3.

Multiple linear regression analyses adjusting for covariates including age, gender, and education (Table 3) indicated that there was no significant association between depression and objective neuropsychological performance (*p*=0.77). There was also no significant association between subjective cognitive complaints and objective neuropsychological performance (*p*=0.52) and the test of moderating effects of depression (*p*=0.69) on the association between subjective cognitive complaints and objective neuropsychological performance (mHDS) was also statistically non-significant. Among the covariates, age was not associated with objective cognitive performance in this sample (*p*=0.08), but being a male was associated with a lower score on the mHDS (B=−1.1558, SE=0.3547, *p*=0.0013) and a greater number of years of education indicated a better score on mHDS (B=0.2846, SE=0.0850, *p*=0.001), controlling for other covariates. We explored whether HIV biomarkers moderated the relationship between subjective cognitive complaints and objective neuropsychological performance, but the results were statistically non-significant (CD4 T cell count [*p*=0.21], plasma HIV RNA [*p*=0.40], % undetectable at <50 copies/mL [*p*=0.36], AIDS status [*p*=0.84], current antiretroviral therapy status [*p*=0.69], duration of treatment [*p*=0.26]).

## Discussion

In the current antiretroviral era, mild neurocognitive impairment is common among PLWH. Cognitive symptoms may be inadequately detected through subjective cognitive complaints and the presence of depressive symptoms may further obscure patient self-report. The purpose of the current study was to describe the relationships among depression, subjective cognitive complaints, and objective neuropsychological performance in PLWH using readily accessible and easily administered instruments, those most likely to be used by frontline healthcare workers. Our study found that while a higher number of depressive symptoms were significantly associated with more subjective cognitive complaints, depression was not associated with objective cognitive performance. Additionally, subjective cognitive complaints and objective cognitive assessments were not associated and were not moderated by depressive symptoms. Among the covariates, age was not associated with subjective cognitive complaints and objective cognitive performance. Males and those with lower education demonstrated worse objective cognitive performance.

The lack of relationship between subjective cognitive complaints and objective cognitive performance among PLWH is consistent with existing literature [11–13]. It is possible that clinically cognitively impaired individuals may be less aware of their actual neurocognitive performance, which may help explain the lack of association between subjective and objective cognitive performance [12]. Inconsistent with our findings, additional literature suggests a significant positive association between subjective cognitive complaints and objective cognitive performance [6–9,29,30], which may be related to the number and types of tests used to assess neuropsychological performance. For example, in their structural equation model, Carter et al. [7] found that cognitive complaints were significantly associated with neuropsychological performance (β=0.39, *p*<01). Carter et al. [7] utilized a battery of subjective and objective neuropsychological tests to create the latent variables-cognitive complaints and neuropsychological skills-that were included in their structural equation model. Based on different findings between Carter et al. [7] and those in our study, it is possible that multiple tests for objective and subjective neuropsychological function may alter the strength and significance of the association between these two variables. Inconsistent findings on the association between subjective cognitive complaints and objective cognitive performance may also be related to the cognitive domains that are assessed as part of the measure of objective cognitive functioning. For example, Bassel et al. [6] reported a significant association between working memory and subjective cognitive complaints (*r*=0.56, *p*<01), but found no significant association between psychomotor functioning and subjective cognitive complaints (*r*=0.31, *p*>05). Similarly, Kamkwalala, et al. [8] found that subjective cognitive complaints were significantly associated with working memory (*r*=0.41, *p*=025), delayed recall (*r*=0.43, *p*=012), and verbal memory (*r*=0.43, *p*=0.012). The mHDS scale used in our study assessed psychomotor speed, verbal recall, and visuo-construction; however, the mHDS total score, not its subscales, was used for assessing the relationship between subjective cognitive complaints and objective functioning. Focus on specific cognitive domains, rather than overall cognitive functioning, may reveal more nuanced relationships between subjective and objective cognitive performance.

Findings from our study showed that subjective cognitive complaints were associated with more symptoms of depression, which is consistent with findings from other studies [29–32,10]. This relationship may exist, as depression is associated with perceived cognitive impairments [33] and depressed individuals may over report subjective cognitive complaints [29]. The issue of overemphasizing cognitive complaints in depressed individuals can also be explained by poor self-insight. The present study also found no association between depressive symptoms and objective neuropsychological performance. Similar results were also found by Claypool et al. [11] who conducted a randomized-placebo controlled study, in which 75 non-demented PLWH were treated for major depression and changes in depression, cognitive complaints, and objective neuropsychological performance were measured at baseline and 12 weeks. Claypool et al. [11] found that cognitive complaints were significantly associated with depressive symptoms, such that greater depression severity was associated with more memory complaints. Additionally, changes in depressive symptoms, not neuropsychological performance, were a significant predictor of cognitive complaints at 12 weeks. The finding from Claypool et al. study [11] and those from our study suggest that cognitive complaints may result from depressed mood and may be less reflective of underlying neuropathological manifestations.

Conversely, additional studies have reported a significant association between depression and objective neuropsychological performance [17,18,34]. The inconsistency in findings between depressive symptoms and objective neuropsychological performance may be attributed to variations in the operationalization of study variables. For example, Fialho et al. [17] analyzed depression categorically based on the Portuguese version of the Beck Depression Scale and may reflect cultural differences in the experience and reporting of symptoms of depression. Moreover, Rourke et al. [18] developed metamemory subtypes that combined memory complaints and verbal learning test scores into a categorical variable. These subtypes were based on clinical experience and may not be appropriate for other study samples. Our findings also show that those with lower education demonstrated worse objective cognitive performance. Research linking educational attainment and cognitive performance is well-established [35–38].

## Limitations

The results of the present study suggest that subjective cognitive complaints may be an inadequate indicator of objective neurocognitive performance and that depressive symptoms may obscure subjective cognitive complaints, but not objective neurocognitive performance. The findings from this study should be interpreted in light of its limitations. First, the participants for this study were recruited from a geographically limited area in Miami, Florida and are likely not representative of the HIV-positive population at large. Second, the measure used to assess for objective cognitive performance (mHDS) is sensitive to the most severe forms of cognitive deficits, as the measure was originally created to detect HIV-associated dementia. Therefore, this measure may have not adequately captured participants with milder levels of cognitive impairment and may have not found significant association between objective and subjective cognitive deficits and moderating effects of depression among those with mild cognitive problems. Third, multicollinearity may account for the significant association between depressive symptoms and subjective cognitive complaints, as both measures of depression (CES-D 10) and subjective cognitive complaints (MOS-HIV) share overlapping items that assess concentration problems. However, there was no significant difference between the results of analyses using the full scale of CES-D 10 and that of revised scale of CES-D 10 without the similar item. Fourth, due to the nature of a secondary data analysis, this study was confined by the instruments and data previously collected by the parent study. We were unable to include other subjective and objective measures of cognitive functioning to determine whether certain measures are more highly correlated with one another. Fifth, the nature of the cross-sectional design limits any causal relationships as well as the generalizability of the study. Also, a meta-analysis on cognition and depression [16] suggests that longitudinally assessed depressive symptoms maybe more closely associated with neuropsychological performance than the one-time point assessment of depression because both affective and cognitive issues in depression can depend on state or trait. The chronicity of depression was not collected and tested in our study. Lastly, there are a host of potential influencing factors on cognitive function among PLWH, including drug use, number of other chronic conditions that affect cognition, antidepressant use, other psychiatric illnesses, and adherence to antiretroviral therapy, that were not accounted for in this secondary analysis and may have influenced study findings.

## Clinical and Research Implications

The results from this study have important implications for healthcare providers of PLWH and for future research. Subjective cognitive complaints may result from a range of depressive symptoms and treatment of these symptoms may assist in decreasing cognitive complaints [11]. Additionally, healthcare providers and researchers should be cognizant that subjective cognitive complaints may be an inadequate indicator of objective neurocognitive performance and should utilize both subjective and objective measures of cognitive performance when making diagnostic decisions.

Future research should investigate the optimal subjective and objective screening tools for neurocognitive deficits among PLWH. Such research should identify which cognitive domains are most likely to be affected by HIV and should account for underlying mental health co-morbidities, such as depression. Screening tool considerations should also include accessibility and ease of administration by frontline workers.

## Conclusion

The current study investigated the relationships among depression, subjective cognitive complaints, and objective neuropsychological performance in PLWH using short measures that could be administered easily by healthcare workers. The results indicate that depression was correlated with subjective cognitive complaints but not with objective neuropsychological performance. Multiple linear regression revealed that objective neuropsychological performance was not statistically associated with subjective cognitive complaints. Implications for clinical practice are that healthcare workers caring for PLWH should not rely exclusively on subjective cognitive complaints but rather assess symptoms of depression and administer a quick objective assessment of cognition to evaluate the source of cognitive symptoms. Furthermore, it may be also important to examine and co-manage symptoms of depression when cognitive complaints are present in PLWH.

## Figures and Tables

**Table 1:** Demographic and clinical characteristics of study sample (N=207)

Variable	N		(Mean ± SD or %)		
Age	207		(47.02 ± 7.43)		
Gender (Male)	96		(46.4)		
**Marital Status**					
Never married/Single	117		(56.5)		
Married/living with partner	20		(9.7)		
Divorced/separated	60		(29.0)		
Widow/widower	10		(4.8)		
**Race/Ethnicity**					
White/non-Hispanic	9		(4.3)		
Hispanic	22		(10.6)		
Black or African American	171		(82.6)		
Other	5		(2.4)		
**Sexual Orientation**					
Homosexual	20		(9.7)		
Heterosexual	169		(81.6)		
Bisexual	16		(7.7)		
Other	1		(0.5)		
Years of Education Completed	207		(11.07 ± 2.10)		
**Employment**					
Unemployed	188		(90.8)		
Employed	19		(9.2)		
Homelessness in past 7 months	39		(18.8)		
Currently taking cART	196		(94.7)		
Undetectable plasma HIV RNA (<50 copies/mL)	101*		(52.6)*		
AIDS status	103		49.8		
**Variable**	**Mean (SD)**		**Median**	**IQR**	**Range**
CES-D10	10.72	(6.63)	10.00	9.00	29.00
MOS-HIV	73.10	(19.31)	75.00	25.00	83.34
mHDS	7.30	(3.14)	7.50	5.50	12.00
Viral load	6254.6	(36990.0)	47.0	600.8	483719.0
CD4 T cell count	407.3	(301.2)	396.5	404.8	1214.0
Duration of cART (months)	34.3	(37.4)	24.0	41.0	204

Note: SD=Standard Deviation; cART=combination Antiretroviral Therapy; CES-D 10=Center for Epidemiological Studies–Depression 10; MOS-HIV=Medical Outcomes Study HIV Health Survey Cognitive Subscale; mHDS=Modified HIV-Dementia Scale;

* data on HIV viral load were available from 192 participants

**Table 2:** Correlation among depression (CES-D 10), subjective cognitive complaints (MOS-HIV), and objective neuropsychological performance (mHDS)

Variables	CES-D10	MOS-HIV	mHDS
CES-D10	------	*r*=−0.66 (CI: −0.73, −0.57)	*r*=−0.04 (CI: −0.18, 0.10)
		*p*<0.0001	*p*<0.0001
MOS-HIV	-----	-----	*r*=0.076 (CI: −0.06, 0.21)
			*p*=0.28

Note: CES-D 10=Center for Epidemiological Studies–Depression 10; MOS-HIV=Medical Outcomes Survey-HIV Cognitive Subscale; mHDS=Modified HIV-Dementia Scale

**Table 3:** Multiple linear regression of subjective cognitive complaints (MOS-HIV) and depression (CES-D 10) on objective neuropsychological performance (mHDS*)

Variables	Unstandardized Estimates		Standardized Estimates	t-stat	*p-value*
	B	SE	Beta		
MOS-HIV	0.0135	0.0209	0.099	0.6458	0.5191
CES-D10	0.0298	0.1034	0.075	0.2880	0.7736
MOS-HIV* CES-D10	−0.0005	0.0013	−0.079	−0.3965	0.6922
Age	−0.0422	0.0241	−0.119	−1.7546	0.0809
Male gender	−1.1558	0.3547	−0.219	−3.2668	0.0013
Years of Education	0.2846	0.0850	0.226	3.3469	0.0010

Note:

* mHDS was transformed as (mHDS) ^ 0.94 prior to the analysis to meet the normality assumption of the regression model.

R^2^=0.12; SE=Standard Error; CES-D 10=Center for Epidemiological Studies–Depression 10; MOS-HIV=Medical Outcomes Survey-HIV Cognitive Subscale; mHDS=Modified HIV-Dementia Scale
